# The Effect of Laparoscopic Radical Hysterectomy Surgical Volume on Oncology Outcomes in Early-Stage Cervical Cancer

**DOI:** 10.3389/fsurg.2021.692163

**Published:** 2021-09-07

**Authors:** Pengfei Li, Jiaqi Liu, Li Wang, Shang Kang, Ying Yang, Jianxin Guo, Jilong Yao, Anwei Lu, Zhonghai Wang, Bin Lin, Zhiqiang Li, Xiaonong Bin, Jinghe Lang, Ping Liu, Chunlin Chen

**Affiliations:** ^1^Department of Obstetrics and Gynecology, Nanfang Hospital, Southern Medical University, Guangzhou, China; ^2^Department of Gynecologic Oncology, Affiliated Cancer Hospital of Zhengzhou University, Zhengzhou, China; ^3^Department of Gynecology, Fourth Hospital of Hebei Medical University, Shijiazhuang, China; ^4^Department of Obstetrics and Gynecology, Xinqiao Hospital, Army Medical University, Chongqing, China; ^5^Department of Obstetrics and Gynecology, Daping Hospital, Army Medical University, Chonqing, China; ^6^Department of Gynecology, Shenzhen Maternity and Child Healthcare Hospital, Shenzhen, China; ^7^Department of Gynecology, Shenzhen Hospital of Southern Medical University, Shenzhen, China; ^8^Department of Gynecology, Shenzhen Sixth People's Hospital, Shenzhen, China; ^9^Department of Obstetrics and Gynecology, China-Japan Friendship Hospital, Beijing, China; ^10^Department of Epidemiology, College of Public Health, Guangzhou Medical University, Guangzhou, China; ^11^Department of Obstetrics and Gynecology, Peking Union Medical College Hospital, Peking Union Medical College, Beijing, China

**Keywords:** cervical cancer, laparoscopy, IB1 stage, surgical volume, oncology outcome

## Abstract

**Purpose:** To examine the association between surgical volume and surgical and oncological outcomes of women with stage IB1 cervical cancer who underwent laparoscopic radical hysterectomy (LRH).

**Methods:** We retrospectively analyzed the oncological outcomes of 1,137 patients with stage IB1 cervical cancer receiving LRH from 2004 to 2016. The surgical volume for each surgeon was defined as low [fewer than 50 surgeries, *n* = 392(34.5%)], mid [51-100 surgeries, *n* = 315(27.7%)], and high [100 surgeries or more, *n* = 430(37.8%)]. Surgical volume-specific survival was examined with Kaplan–Meier analysis, multivariable analysis, and propensity score matching.

**Results:** The operative times of the high-volume group (227.35 ± 7.796 min) were significantly shorter than that of the low- (272.77 ± 4.887 min, *p* < 0.001) and mid-volume (255.86 ± 4.981 min, *p* < 0.001) groups. Blood loss in the high-volume group (169.42 ± 8.714 ml) was significantly less than that in the low-volume group (219.24 ± 11.299 ml, *p* = 0.003). The 5-year disease-free survival (DFS) and overall survival (OS) in the low-volume, mid-volume, and high-volume groups were similar (DFS: 91.9, 86.7, and 89.2%, *p* = 0.102; OS: 96.4, 93.5, and 94.2%, *p* = 0.192). Multivariable analysis revealed surgical volume was not an independent risk factor for OS or DFS. The rate of intraoperative and postoperative complications was similar among the three groups (*p* = 0.210).

**Conclusions:** Surgical volume of LRH may not be a prognostic factor for patients with stage IB1 cervical cancer. Surgery at high-volume surgeon is associated with decreased operative time and blood loss.

## Introduction

Radical hysterectomy (RH) with pelvic lymphadenectomy is the conventional treatment for early-stage cervical cancer (1). The laparoscopic technique was first reported for RH in 1992 (2), and since then, minimally invasive surgery has become a common surgical approach because of the advantages of less intraoperative blood loss, fewer operative complications, and shorter hospital stay when compared with traditional open surgery (3–7). However, the laparoscopic approach to cervical cancer (LACC) trial (8), a high-quality international multicenter randomized controlled trial, reported that laparoscopic radical hysterectomy (LRH) was closely related to worse oncology outcomes compared with abdominal radical hysterectomy (ARH). Consequently, the National Comprehensive Cancer Network (NCCN) guidelines recommend laparotomy as the standard approach for a RH for cervical cancer (9).

Several recent retrospective studies have also demonstrated that minimally invasive RH was associated with shorter OS and DFS rates than that of ARH (9–12). But these studies failed to analyze the reasons. The surgical experience of surgeons is often considered to affect the outcomes of surgery. So, the surgical experience of surgeons may be one of the reasons influencing the oncology outcomes of LRH (13–15). However, there are limited data specifically analyzing the impact of the experience of surgeons in LRH, and most of their main concern was surgical skills or short-term effects (16–18), and little concentration was focused on long-term survival outcomes. Most of these studies involved single centers and surgeons, with few cases included (17, 19, 20).

Therefore, based on the large clinical diagnosis and treatment database of cervical cancer in the mainland China (Four C) database, we explored the association between the LRH volume of surgeons and long-term oncological outcomes of women with early-stage cervical cancer who underwent LRH. We divided the patients into low-, medium-, and high-surgical volume groups according to the surgical experience of surgeons and compared the 5-year overall survival rates and disease-free survival rates of patients with stage IB1 cervical cancer among the three groups.

## Methods

### Data Source

The data of this study derive from the clinical diagnosis and treatment for cervical cancer in mainland China (Four C) database, a cervical cancer-specialized disease database (*n* = 46,313) that covers consecutive patients with cervical cancer in 37 hospitals in mainland China treated between January 2004 and December 2016. The Ethics Committee of Nanfang Hospital, Southern Medical University, approved this retrospective study (ethics number NFEC-2017-135). Written informed consent was waived by the ethics committee, as the information of human medical documents was retrospectively gathered and analyzed and patient data were unidentifiable in this study. The identifier of the clinical trial is CHiCTR1800017778 (International Clinical Trials Registry Platform Search Port, http://apps.who.int/trialsearch/).

Using standardized data collection and quality control procedures, trained gynecological oncology staff collected the clinical data from patient files and the medical record management system in the hospitals. The details of the data sources and methods were the same as those previously reported (21, 22). For patients undergoing surgical treatment, the collected data, including demographic details, preoperative examination results, surgical information, pathological results, preoperative and postoperative adjuvant treatment details, complications, hospitalization time, expenses, and follow-up, contained almost all the information during the treatment of cervical cancer. In this database, the International Federation of Gynecology and Obstetrics (FIGO) stage was recorded and corrected by tumor size according to the FIGO 2009 staging system. Tumor size was evaluated by pathological records. To ensure the accuracy of the collected data, two uniformly trained staff members used EpiData software (EpiData Association, Odense M, Denmark) to input and proofread the same data from each hospital.

All follow-up procedures were carried out by trained gynecological oncology staff at each center to keep the personal data of patients confidential and to provide disease-management guidance. The follow-up information, including survival status, time of death, recurrence time, recurrence site, and treatment after recurrence, was gathered through the return visit system or telephone follow-up. Oncological outcomes were assessed according to the recorded information, and the last day of the return visit or telephone follow-up was defined as the last follow-up. The follow-up rate of oncological outcomes in this database is 72.7%.

### Inclusion and Exclusion Criteria

The inclusion criteria were as follows: (1) age > 18 years old; (2) FIGO 2009 stage IB1; (3) squamous cell carcinoma, adenocarcinoma, or adenosquamous cell carcinoma; (4) Q-M type B2 or type C2 hysterectomy + pelvic lymphadenectomy; (5) available survival outcomes; (6) no preoperative treatments; and (7) standard postoperative adjuvant treatment after the operation.

The exclusion criteria were as follows: (1) pregnancy; (2) cervical stump cancer; (3) combined with other malignant tumors; and (4) history of pelvic surgery.

### Definitions and Outcome Measures

In this study, patients treated by surgeons with different LRH experiences were divided into three groups (19): low-volume surgeons (<51), mid-volume surgeons (51-100), and high-volume surgeons (>100). Outcome measures included operative technique (operative time and blood loss) and oncological outcome (5-year OS and DFS).

The OS was defined as the time from the date of surgery to the date of death from any cause. DFS was defined as the time from the date of surgery to the date of disease recurrence or death from cervical cancer, and patients with no evidence of recurrence or death were censored at the date of the last follow-up or return visit.

According to FIGO guidelines (23), standard postoperative treatment was defined by any one pathological high-risk factor (positive para-aortic or pelvic nodes, parametrial extension, and positive margins) or any two or more pathological medium-risk factors (tumor size > 4 cm, lymph-vascular space invasion (LVSI), stromal invasion), who received radiotherapy or radiochemotherapy or those with no postoperative high-risk factor and one or more medium-risk factors, who did not receive postoperative radiotherapy or radiochemotherapy.

Complications were classified into intraoperative complications and postoperative complications. Intraoperative complications included ureteral injury, bladder injury, bowel injury, vascular injury, and obturator nerve injury. Postoperative complications within 2 years of surgery included bowel obstruction, pelvic hematoma, hemorrhage, vesicovaginal fistula, ureterovaginal fistula, ureteral fistula, rectovaginal fistula, venous thromboembolism, and chylous leakage (24).

### Statistical Methods

All statistical procedures were processed with Statistical Product and Service Solutions (SPSS) 23.0 statistical software (SPSS, Inc., Chicago, IL, USA). Between-group differences in the baseline characteristics were assessed through independent two-sample *t*-tests or Pearson's chi-squared test. Quantitative data are shown as the mean ± SD (x ± s), and nominal-scale data are shown as percentages (%). The 5-year OS and DFS rates of LRH and ARH were calculated and compared using the Kaplan–Meier curve and the log-rank test. A Cox proportional hazards model was used to estimate hazard ratio (HR)s and 95% CIs for the effect of treatment on 5-year OS and DFS; known factors that may affect the oncological outcome of cervical cancer were included in this multivariate model to adjust for case-mix, including age, hospital type, region, city class, finance, year, Q-M type, histology, LVSI, stromal invasion, uterine corpus invasion, parametrial tumor involvement, surgical margin invasion, lymph node metastasis, preoperative treatment condition, and postoperative adjuvant treatment. Propensity score matching (PSM, 1:1) was used to balance differences in the data analysis; the variables included were the same as those above. A *P*-value < 0.05 from two-sided tests was regarded as significant.

## Results

**Figure 1:**
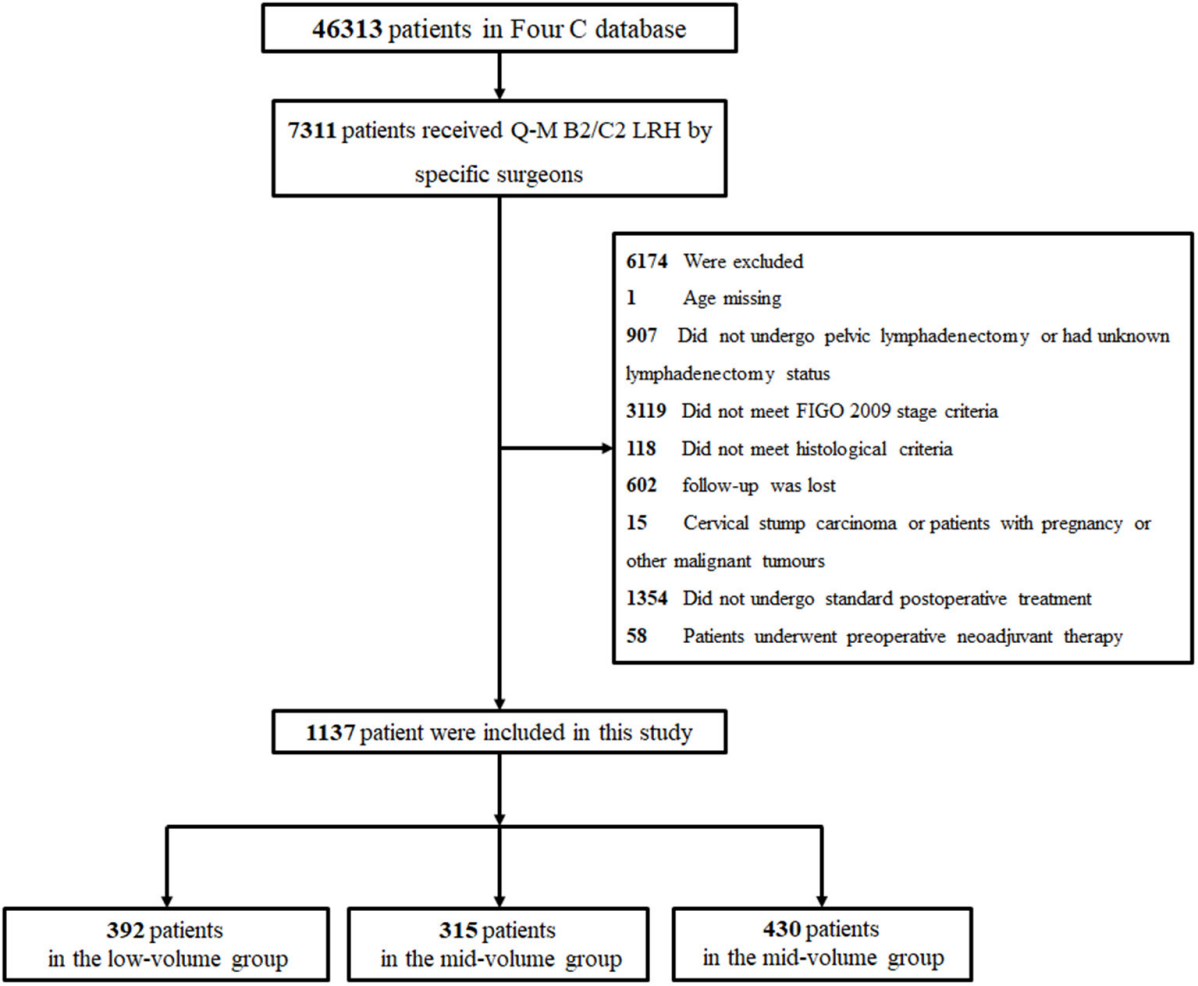
Flowchart of patients included in the analysis.

Among the 46,313 patients described in the database, 1,137 were included in this study (392 patients in the low-volume group, 315 patients in the mid-volume group, and 430 patients in the high-volume group). The flow diagram of recruitment and exclusion is illustrated in Figure 1. The median follow-up time was 42 months. The clinicopathologic characteristics of the three groups are provided in Table 1. The high-volume group comprised more patients from the north and southwest region and second-tier cities (*p* < 0.001) and less patients from the general hospital (*p* = 0.010) than the other groups. The low-volume group comprised less patients from the countryside than the other groups (*p* = 0.018). Women in the high-volume group were more likely to receive Querleu-Morrow (Q-M) type C2 RH compared with the other two groups (*p* < 0.001). The patients in the high-volume group were more likely to have LVSI (*p* = 0.026) compared with the women in the other two groups; those in the mid- and high-volume groups were more likely to have deep stromal than were those in the low-volume group (*p* = 0.008). No significant differences were found among the three groups in the number of lymph nodes removed, histological types, uterine infiltration, parametrial involvement, surgical margin invasion, lymph node metastasis, and postoperative treatment (*p* > 0.05).

**Table 1:** Clinical and pathological characteristics of the three groups.

	**Low**		**Mid**		**High**		***P***
	**(** ***n*** **=** **392, %)**		**(** ***n*** **=** **315, %)**		**(** ***n*** **=** **430, %)**		
Age (y)	46.68 ± 9.175		47.35 ± 9.281		47.25 ± 9.312		0.569
Hospital type							**0.010**
General hospital	210	53.60%	201	63.80%	220	51.20%	
Tumor Hospital	138	35.20%	91	28.90%	162	37.70%	
Women-children specialty hospital	44	11.20%	23	7.30%	48	11.20%	
Region							** <0.001**
North China	116	29.60%	82	26.00%	207	48.10%	
South China	141	36.00%	123	39.00%	58	13.50%	
Central China	42	10.70%	17	5.40%	26	6.00%	
East China	88	22.40%	58	18.40%	32	7.40%	
Southwest China	5	1.30%	35	11.10%	107	24.90%	
City class							** <0.001**
First-tier	140	35.70%	65	20.60%	21	4.90%	
Second-tier	176	44.90%	152	48.30%	318	74.00%	
Third-tier and above	76	19.40%	98	31.10%	91	21.20%	
Economics							**0.018**
Countryside	205	52.30%	191	60.60%	246	57.20%	
Urban	141	36.00%	107	34.00%	153	35.60%	
Unknown	46	11.70%	17	5.40%	31	7.20%	
Year							**0.036**
2005-2013	117	29.80%	83	26.30%	154	35.80%	
2014-2015	149	38.00%	134	42.50%	167	38.80%	
2016	126	32.10%	98	31.10%	109	25.30%	
Q-M Type							** <0.001**
QM-B2	251	64.00%	101	32.10%	43	10.00%	
QM-C2	141	36.00%	214	67.90%	387	90.00%	
Number of lymph nodes removed	21.97 ± 9.917	23.16 ± 10.934	21.42 ± 11.596	0.096			
Histology							0.108
Squamous	338	86.20%	262	83.20%	384	89.30%	
Adenocarcinoma	51	13.00%	49	15.60%	40	9.30%	
Adenosquamous	3	0.80%	4	1.30%	6	1.40%	
LVSI							**0.026**
No	346	88.30%	258	81.90%	354	82.30%	
Yes	46	11.70%	57	18.10%	76	17.70%	
Deep stromal invasion							**0.008**
No	250	63.80%	179	56.80%	276	64.20%	
Yes	81	20.70%	95	30.20%	112	26.00%	
Unreported	61	15.60%	41	13.00%	42	9.80%	
Uterine infiltration							0.129
No	380	96.90%	297	94.30%	405	94.20%	
Yes	12	3.10%	18	5.70%	25	5.80%	
Parametrial involvement							0.257
No	389	99.20%	310	98.40%	428	99.50%	
Yes	3	0.80%	5	1.60%	2	0.50%	
Surgical margin invasion							0.471
No	389	99.20%	311	98.70%	428	99.50%	
Yes	3	0.80%	4	1.30%	2	0.50%	
Lymph node metastasis							0.196
No	350	89.30%	276	87.60%	366	85.10%	
Yes	42	10.70%	39	12.40%	64	14.90%	
Adjuvant therapy							0.157
None	327	83.40%	244	77.50%	332	77.20%	
Chemotherapy alone	0	0.00%	0	0.00%	0	0.00%	
Radiotherapy alone	4	1.00%	6	1.90%	10	2.30%	
Chemoradiotherapy	61	15.60%	65	20.60%	88	20.50%	

### Surgical Outcomes

The operative times in low-, mid-, and high-volume groups were 272.77 ± 4.887 min, 255.86 ± 4.981 min, and 227.35 ± 7.796 min, respectively (*p* < 0.001). There was no significant difference in operative time between the low- and mid-volume groups (*p* = 0.096). While the operative times of the high-volume group were significantly shorter than the low- and mid-volume groups (all *p* < 0.001). Among the 1,137 patients, the average intraoperative blood loss was 219.24 ± 11.299 ml, 198.08 ± 10.595 ml, and 169.42 ± 8.714 ml in the low-, mid-, and high-volume groups, respectively (*p* = 0.002). There was no significant difference between the mid-volume group and the low- (*p* = 0.258) and high-volume groups (*p* = 0.058), but blood loss in the high-volume group was significantly less than that in the low-volume group (*p* = 0.003).

### Results of Complications

In the total study population, complications occurred in 18(4.6%), 14(4.4%), and 30(7.0%) patients in the low-, mid-, and high-volume group, respectively. The complication data are shown in Table 2. The rate of any one complication refers to the incidence of one or more complications in a patient, which had similar injury rates among the three groups (*p* = 0.210); moreover, the three groups had similar rates of intraoperative (*p* = 0.745) and postoperative (*p* = 0.145) complications.

**Table 2:** Intraoperative and postoperative complications among low-, mid-, and high-volume group.

	**Low**		**Mid**		**High**		***P***
	***n***	**%**	***N***	**%**	***n***	**%**	
Any 1 complication	18	4.60%	14	4.40%	30	7.00%	0.210
Intraoperative complication	4	1.00%	4	1.30%	7	1.60%	0.745
Vascular injury	0	0.00%	0	0.00%	0	0.00%	-
Ureteral injury	1	0.30%	3	1.00%	5	1.20%	0.317
Obturator nerve injury	1	0.30%	0	0.00%	0	0.00%	0.386
Bladder injury	2	0.50%	1	0.30%	1	0.20%	0.792
Bowel injury	0	0.00%	0	0.00%	0	0.00%	-
Postoperative complication	14	3.60%	10	3.20%	25	5.80%	0.145
Vesicovaginal fistula	3	0.80%	1	0.30%	1	0.20%	0.478
Ureterovaginal fistula	6	1.50%	4	1.30%	6	1.40%	0.958
Rectovaginal fistula	0	0.00%	0	0.00%	3	0.70%	0.084
Venous thromboembolism	1	0.30%	4	1.30%	9	2.10%	0.058
Bowel obstruction	2	0.50%	2	0.60%	3	0.70%	0.942
Chylous leakage	1	0.30%	0	0.00%	2	0.50%	0.473
Pelvic haematoma	0	0.00%	0	0.00%	1	0.20%	0.439
Haemorrhage	1	0.30%	1	0.30%	1	0.20%	0.975

Among the intraoperative complications, the main complication was ureteral injury (9/1137, 0.79%). The rate of ureteral injury was higher in the high-volume group than in the low- and mid-volume groups, but the difference among them was insignificant (*p* = 0.317); the obturator nerve and bladder injury rates were similar among the three groups (*p* > 0.050). There were no cases of vascular injury or bowel injury in either group.

Among the postoperative complications, the main complications were ureterovaginal fistula (16/1137, 1.41%), venous thromboembolism (14/1137, 1.23%), and bowel obstruction (7/1137, 0.61%). The three groups had similar rates of vesicovaginal fistula, ureterovaginal fistula, rectovaginal fistula, venous thromboembolism, bowel obstruction, chylous leakage, pelvic hematoma, and active abdominal and pelvic bleeding (*p* > 0.05).

### Oncological Outcomes

**Figure 2:**
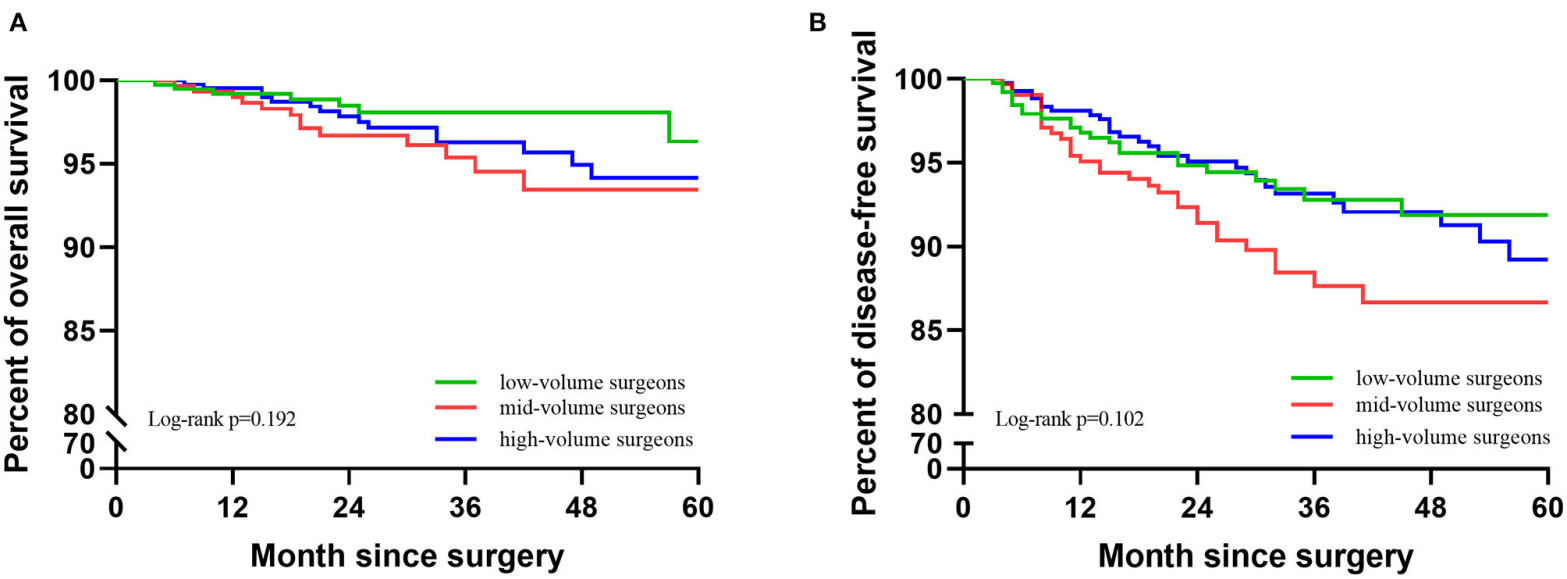
Kaplan-Meier survival curves of the three groups. **(A)** KM of OS of the three groups. **(B)** KM of DFS of the three groups.

In 1,137 cases, 35 cases died and 83 cases recurred. In the low-, mid-, and high-volume groups, the 5-year DFS was 91.9, 86.7, and 89.2%; the 5-year OS was 96.4, 93.5, and 94.2%; there was no significant difference between the three groups (DFS: *p* = 0.102; OS: *p* = 0.192). The Kaplan–Meier survival curves are shown in Figure 2. In the multivariable analysis, adjusting for the case-mix, patients in different volume groups were not an independent worse prognostic factor for a worse 5-year DFS or OS (DFS: *p* = 0.239;OS: *p* = 0.206, Table 3).

**Table 3:** Association of surgical volume and survival in cervical cancer.

	**Disease-Free Survival**			**Overall Survival**				
**Project**	***P***	**HR**	**95%CI**		***P***	**HR**	**95%CI**	
**Before group matching**								
Low-volume group	0.206				0.239			
Mid-volume group	0.084	2.515	0.884	7.157	0.126	1.596	0.877	2.902
High-volume group	0.402	1.658	0.508	5.412	0.763	1.116	0.547	2.277
**After group matching**								
Low- vs. Mid-	0.051	4.526	0.994	20.622	0.120	1.800	0.859	3.775
Low- vs. High-	0.839	11849135.200	0.000	1.438E + 75	0.426	2.018	0.359	11.354
Mid- vs. High-	0.426	0.646	0.220	1.894	0.477	0.767	0.369	1.593

**Figure 3:**
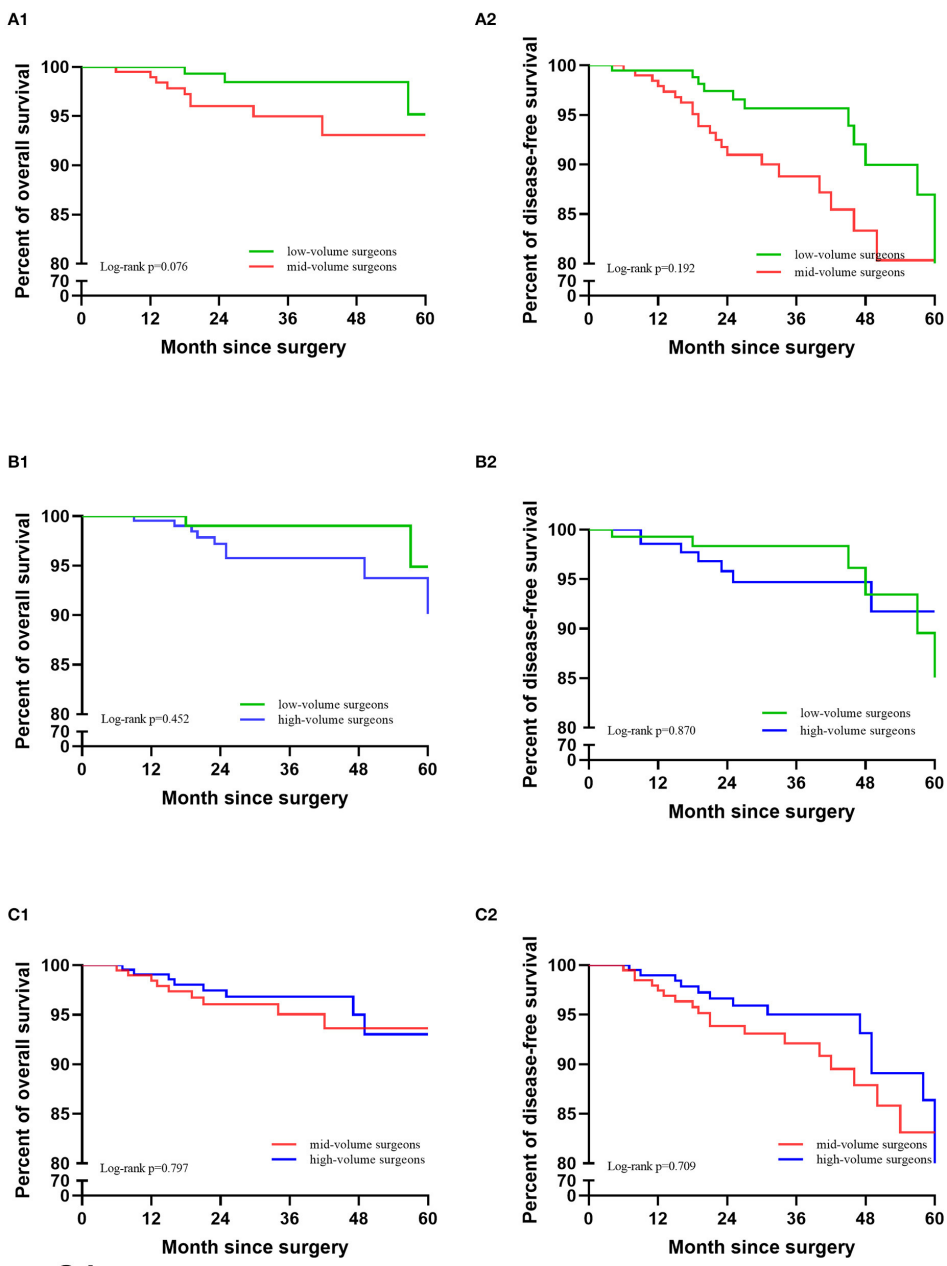
Kaplan-Meier survival curves after PSM matching. **(A)** Low-volume group vs. mid-volume group. **(B)** Low-volume group vs. high-volume group. **(C)** Mid-volume group vs. high-volume group.

After 1:1 PSM, the clinicopathological characteristics were well-balanced (Table 4). For the low-volume group vs. the mid-volume group, including 201 patients in each, there was no significant difference in the 5-year DFS or OS (DFS: 91.0 vs. 85.8%, *p* = 0.192; OS: 95.2 vs. 93.1%; *p* = 0.076). The Kaplan-Meier survival curves are shown in Figure 3. In Cox regression analysis adjusting for the above factors, the volume of surgery was not an independent risk factor for DFS and OS (DFS: *p* = 0.120; OS: *p* = 0.051).

**Table 4:** Clinical and pathological characteristics among three groups after PSM matching.

	**Low**		**Mid**		***P***	**Low**		**High**		***P***	**Mid**		**High**		***P***
	**(** ***n*** **=201,%)**		**(** ***n*** **=** **201,%)**			**(** ***n*** **=** **145,%)**		**(** ***n*** **=** **145,%)**			**(** ***n*** **=** **202,%)**		**(** ***n*** **=** **202,%)**		
Age (y)	47.78 ± 9.524		47.14 ± 9.154		0.345	46.92 ± 9.211		47.21 ± 9.132		0.897	47.46 ± 9.819		47.33 ± 9.241		0.385
Hospital type					0.916					0.602					0.773
General hospital	123	61.20%	119	59.20%		88	60.70%	96	66.20%		119	58.90%	112	55.40%	
Tumor Hospital	71	35.30%	75	37.30%		52	35.90%	44	30.30%		61	30.20%	67	33.20%	
Women-children specialty hospital	7	3.50%	7	3.50%		5	3.40%	5	3.40%		22	10.90%	23	11.40%	
Region					0.597					0.315					0.854
North China	63	31.30%	64	31.80%		71	49.00%	73	50.30%	.	81	40.10%	84	41.60%	
South China	67	33.30%	65	32.30%		36	24.80%	23	15.90%		47	23.30%	44	21.80%	
Central China	19	9.50%	15	7.50%		18	12.40%	21	14.50%		8	4.00%	11	5.40%	
East China	47	23.40%	46	22.90%		15	10.30%	19	13.10%		34	16.80%	28	13.90%	
Southwest China	5	2.50%	11	5.50%		5	3.40%	9	6.20%		32	15.80%	35	17.30%	
City class					0.14					0.187					0.814
First-tier	52	25.90%	44	21.90%		33	22.80%	21	14.50%		25	12.40%	21	10.40%	
Second-tier	114	56.70%	106	52.70%		84	57.90%	91	62.80%		125	61.90%	129	63.90%	
Third-tier and above	35	17.40%	51	25.40%		28	19.30%	33	22.80%		52	25.70%	52	25.70%	
Economics					0.906					0.408					0.943
Countryside	123	61.20%	120	59.70%		93	64.10%	90	62.10%		124	61.40%	127	62.90%	
Urban	66	32.80%	67	33.30%		44	30.30%	41	28.30%		68	33.70%	66	32.70%	
Unknown	12	6.00%	14	7.00%		8	5.50%	14	9.70%		10	5.00%	9	4.50%	
Year					0.53					0.932					0.586
2005-2013	61	30.30%	51	25.40%		47	32.40%	46	31.70%		69	34.20%	65	32.20%	
2014-2015	76	37.80%	83	41.30%		51	35.20%	54	37.20%		76	37.60%	86	42.60%	
2016	64	31.80%	67	33.30%		47	32.40%	45	31.00%		57	28.20%	51	25.20%	
Q-M Type					0.762					0.775					0.072
QM-B2	84	41.80%	87	43.30%		32	22.10%	30	20.70%		44	21.80%	30	14.90%	
QM-C2	117	58.20%	114	56.70%		113	77.90%	115	79.30%		158	78.20%	172	85.10%	
Number of lymph nodes removed	18.67 ± 9.765	22.71 ± 10.564	0.454	21.49 ± 10.808	23.11 ± 10.093	0.697	22.48 ± 10.347	23.31 ± 10.778	0.808						
Histology					1					1					0.833
Squamous	172	85.60%	172	85.60%		130	89.70%	130	89.70%		171	84.70%	175	86.60%	
Adenocarcinoma	28	13.90%	28	13.90%		14	9.70%	14	9.70%		27	13.40%	24	11.90%	
Adenosquamous	1	0.50%	1	0.50%		1	0.70%	1	0.70%		4	2.00%	3	1.50%	
LVSI					0.663					0.866					0.793
No	175	87.10%	172	85.60%		125	86.20%	124	85.50%		168	83.20%	166	82.20%	
Yes	26	12.90%	29	14.40%		20	13.80%	21	14.50%		34	16.80%	36	17.80%	
Deep stromal invasion					0.821					0.521					0.896
No	125	62.20%	130	64.70%		101	69.70%	92	63.40%		122	60.40%	125	61.90%	
Yes	46	22.90%	45	22.40%		30	20.70%	35	24.10%		54	26.70%	54	26.70%	
Unreported	30	14.90%	26	12.90%		14	9.70%	18	12.40%		26	12.90%	23	11.40%	
Uterine infiltration					0.586					0.702					1
No	195	97.00%	193	96.00%		141	97.20%	142	97.90%		193	95.50%	193	95.50%	
Yes	6	3.00%	8	4.00%		4	2.80%	3	2.10%		9	4.50%	9	4.50%	
Parametrial involvement					0.253					1					0.562
No	199	99.00%	196	97.50%		144	99.30%	144	99.30%		200	99.00%	201	99.50%	
Yes	2	1.00%	5	2.50%		1	0.70%	1	0.70%		2	1.00%	1	0.50%	
Surgical margin invasion					0.562					0.316					0.317
No	200	99.50%	199	99.00%		145	100.00%	144	99.30%		201	99.50%	202	100.00%	
Yes	1	0.50%	2	1.00%		0	0.00%	1	0.70%		1	0.50%	0	0.00%	
Lymph node metastasis				0.874					0.527						0.642
No	178	88.60%	179	89.10%		123	84.80%	119	82.10%		180	89.10%	177	87.60%	
Yes	23	11.40%	22	10.90%		22	15.20%	26	17.90%		22	10.90%	25	12.40%	
Adjuvant therapy				0.991					0.607						0.645
None	163	81.10%	164	81.60%		115	79.30%	108	74.50%		161	79.70%	160	79.20%	
Chemotherapy alone	0	0.00%	0	0.00%		0	0.00%	0	0.00%		0	0.00%	0	0.00%	
Radiotherapy alone	2	1.00%	2	1.00%		2	1.40%	2	1.40%		4	2.00%	7	3.50%	
Chemoradiotherapy	36	17.90%	35	17.40%		28	19.30%	35	24.10%		37	18.30%	35	17.30%	

There were also no significant differences in OS and DFS between the low-volume and high-volume groups, including 145 patients in each (DFS: 95.5 vs. 94.2%, *p* = 0.870; OS: 94.9 vs. 96.3%; *p* = 0.452). The Kaplan-Meier survival curves are shown in Figure 3. After eliminating the case-mix factors by Cox proportional hazards model analysis, the volume of surgery was not an independent risk factor for DFS and OS (DFS: *p* = 0.426; OS: *p* = 0.839).

Between the mid-volume and high-volume groups, with 202 patients in each group, there were no significant differences in DFS and OS (DFS: 88.5 vs. 83.1%, *p* = 0.709; OS: 93.6 vs. 92.5%; *p* = 0.797). The Kaplan-Meier survival curves are shown in Figure 3. After eliminating the case-mix factors by Cox regression analysis, the surgical volume of surgeons was not an independent risk factor for DFS and OS (DFS: *p* = 0.477; OS: *p* = 0.426).

## Discussions

In this study, we divided patients into low-, mid-, and high-volume groups according to the total volume of LRH experience of surgeons and analyzed their effects on long-term oncological outcomes (DFS and OS), surgical outcomes (operative time and blood loss), and operative complications through a multisurgeon, large sample, and multicenter study. Key findings are that the three groups showed similar 5-year DFS and OS rates in the unadjusted and adjusted analysis, which means that the surgical volume of surgeons of LRH may not affect the long-term survival outcomes for patients with stage IB1 cervical cancer. We also found that intraoperative blood loss decreased and operative time was significantly shortened with an increase in surgical volume, while the incidence rate of operative complications among the three groups was similar.

Following the LACC trial, several retrospective studies have also demonstrated that minimally invasive RH was associated with worse oncological outcomes than that of ARH. There have been several changes to the recommendations for LRH in relevant international guidelines, and these changes state that patients must be informed of the LACC results. Both NCCN (9) and European Society of Gynaecological Oncology (ESGO) (25, 26) stated that open surgery is the standard method for cervical cancer surgery. But these studies rarely analyzed the reasons of poor oncological outcomes of LRH.

Surgical experience may be one of the reasons influencing the oncology outcomes of LRH (13–15). But there are few studies analyzing the long-term survival impact of surgical experience in LRH. This large multicenter retrospective cohort study complements the evidence that surgical experience may not be a risk factor for LRH. The results of this study are similar to that of a recent retrospective study taken by scholar Chong et al. (13). They found similar 5-year OS and DFS between the first 50 LRH patients and the second 50 LRH patients performed by the same doctor and concluded that the increase in LRH experience may have no effect on oncological outcomes. They focused on the analysis of a single doctor, with good consistency, but the sample size was small. Our study was a multicenter, multisurgeon, large-sample analysis, and Cox regression analysis, and PSM were used to balance the case-mix factors; thus, the results are more convincing.

There are also studies with different results. In a multicenter study in Japan, Matsuo et al. (14) analyzed the oncological outcomes of 5,964 patients who underwent RH in 116 institutions and considered that high-volume hospitals may be associated with the risk of local recurrence and the improvement of survival rate. However, the surgical approaches were not included in the multivariable analysis because of the unavailable information. The surgical volume of institutions does not reflect the experience of surgeons. There may exist low-volume surgeons in high-volume institutions. Similarly, low-volume institutions may also have high-volume surgeons. This method may not exactly demonstrate how the surgical experience could affect the oncological outcomes. Our study makes up for the deficiency of Matsuo et al. We limited our inclusion criteria of patients who underwent LRH, and we focused on the specific experience of surgeons, which could better represent the effect of surgical experience on the efficacy of cervical cancer.

Laparoscopic surgery involves new technologies and equipment, with the surgical field being transformed from traditional three-dimensional open surgery to two-dimensional laparoscopic surgery. The increase in surgical experience means the repeated practice of surgical skills. In general, theoretical and practical research results confirm that laparoscopic surgery skills can be improved with an increase in surgical volume. Many previous learning curve studies of LRH have proved it (16–18). A meta-analysis to investigate the relationship between the number of gynecology surgeries and surgical outcomes has been conducted, and it is believed that intraoperative and postoperative complications are significantly reduced with high-volume surgeons (27). In the study of Chong et al. (13), it is undeniable that the operative time, hospital stay, time to restore normal residual urine volume, blood loss, intraoperative, and postoperative complications are significantly reduced and that the number of lymph nodes acquired is increased with an increase in the surgical experience. In our study, the surgical skills were significantly improved in the more experienced group, which is similar to Chong et al. Although the statistical difference is not significant among the three groups, the high-volume group had the trend of the higher incidence rate of complication. This may be because of the higher proportion of Q-M type C2 in the high-volume group.

However, we acknowledge several limitations in this study. First, this was a retrospective study with confounding factors. For example, in the high-volume group, the proportion of patients with LVSI and deep stromal invasion was higher than that in the mid- and low-volume groups. Nonetheless, we attempted to balance these differences through PSM. Second, the patient files and medical records among hospitals might be different, leading to the absence of clinical data, such as the information of the usage of a uterine manipulator, previous surgery, the surgical complexity score, or American Society of Anesthesiologists (ASA) score. Third, although our study covered a total of 46,313 cases of cervical cancer inpatients in 37 hospitals in most of China, it did not cover all regions nationwide. Fourth, we excluded cases that might affect the difficulty of surgery, such as the history of pelvic surgery, preoperative chemotherapy, or pelvic radiotherapy. However, anatomical variation, pelvic adhesion, and other parameters that may affect the difficulty of surgery are not available in our database. Fifth, some patients with endometrial cancer might receive LRH, but this database was a cervical cancer-specialized disease database, so we did not consider the LRH volume of patients with endometrial cancer. Sixth, the ASA score of patients may influence the oncological outcomes, but the ASA score was not available in our database, so we did not consider the ASA score in this study.

Despite the above flaws, based on the large sample size of the study, we conclude the following clinical significance. First, we consider that surgical skills can be improved with rich experience. Shortening of the operative time and the reduction of intraoperative blood loss can minimize harm to patients during the operation. Second, surgical experience may not be the factor that affects the long-term oncological outcomes of LRH. Therefore, we need to further explore the limitations of laparoscopic technology itself.

## Data Availability Statement

The raw data supporting the conclusions of this article will be made available by the authors, without undue reservation.

## Ethics Statement

The studies involving human participants were reviewed and approved by the Ethics Committee of Nanfang Hospital, Southern Medical University. The patients/participants provided their written informed consent to participate in this study.

## Author Contributions

CC, PL, LW, and JLa: study design. PL, JLi, and ZL: literature search. PL and JLi: figures. PL, CC, and XB: data analysis. PL and ZL: data collection. PL and JLi: drafting of the manuscript. CC: obtained funding. CC, LW, PL, and JLa: supervision. All authors data interpretation and approved the final version of the study.

## Funding

This study was initially funded by the National Science and Technology Support Program of China (2014BAI05B03), the National Natural Science Fund of Guangdong (2015A030311024), the Science and the Science and Technology Plan of Guangzhou (158100075), the Guangdong Medical Science and Technology Research Fund Project (A2020077), the Basic and Applied Basic Research Fund of Guangdong Province (2019A1515110337), the Chinese Postdoctoral Science Foundation (2019M660207), and the Nanfang Hospital President Fund (2019C005).

## Conflict of Interest

The authors declare that the research was conducted in the absence of any commercial or financial relationships that could be construed as a potential conflict of interest.

## Publisher's Note

All claims expressed in this article are solely those of the authors and do not necessarily represent those of their affiliated organizations, or those of the publisher, the editors and the reviewers. Any product that may be evaluated in this article, or claim that may be made by its manufacturer, is not guaranteed or endorsed by the publisher.
